# Supplementary material for: Iterative improvement in the automatic modular design of robot swarms
Source: PeerJ Comput Sci. 2020 Dec 7;6:e322. doi: 10.7717/peerj-cs.322 (PMC7924708; doi:10.7717/peerj-cs.322)
Supplement: Supplemental Information 3 [file peerj-cs-06-322-s003.zip › argos3/doc/api/standalone/a00542_source.html]

ARGoS: plugins/robots/generic/simulator/range\_and\_bearing\_default\_actuator.cpp Source File


- Main Page
- Related Pages
- Namespaces
- Classes
- Files

- File List
- File Members

# plugins/robots/generic/simulator/range\_and\_bearing\_default\_actuator.cpp

Go to the documentation of this file.

```
00001 
00007 #include "range_and_bearing_default_actuator.h"
00008 #include <argos3/core/simulator/entity/composable_entity.h>
00009 #include <argos3/core/simulator/simulator.h>
00010 
00011 namespace argos {
00012 
00013    /****************************************/
00014    /****************************************/
00015 
00016    void CRangeAndBearingDefaultActuator::SetRobot(CComposableEntity& c_entity) {
00017       m_pcRangeAndBearingEquippedEntity = &c_entity.GetComponent<CRABEquippedEntity>("rab");
00018       m_pcRangeAndBearingEquippedEntity->Enable();
00019       m_cData.Resize(m_pcRangeAndBearingEquippedEntity->GetMsgSize());
00020    }
00021 
00022    /****************************************/
00023    /****************************************/
00024 
00025    void CRangeAndBearingDefaultActuator::Update() {
00026       m_pcRangeAndBearingEquippedEntity->SetData(m_cData);
00027    }
00028 
00029    /****************************************/
00030    /****************************************/
00031 
00032    void CRangeAndBearingDefaultActuator::Reset() {
00033       m_cData.Zero();
00034    }
00035 
00036    /****************************************/
00037    /****************************************/
00038 
00039    REGISTER_ACTUATOR(CRangeAndBearingDefaultActuator,
00040                      "range_and_bearing", "default",
00041                      "Carlo Pinciroli [ilpincy@gmail.com]",
00042                      "1.0",
00043                      "The range and bearing actuator.",
00044                      "This actuator allows robots to perform situated communication, i.e., a form of\n"
00045                      "wireless communication whereby the receiver also knows the location of the\n"
00046                      "sender with respect to its own frame of reference.\n"
00047                      "This actuator allows a robot to send messages. To receive messages, you need\n"
00048                      "the range-and-bearing sensor.\n"
00049                      "To use this actuator, in controllers you must include the\n"
00050                      "ci_range_and_bearing_actuator.h header.\n\n"
00051                      "REQUIRED XML CONFIGURATION\n\n"
00052                      "  <controllers>\n"
00053                      "    ...\n"
00054                      "    <my_controller ...>\n"
00055                      "      ...\n"
00056                      "      <actuators>\n"
00057                      "        ...\n"
00058                      "        <range_and_bearing implementation=\"default\" />\n"
00059                      "        ...\n"
00060                      "      </actuators>\n"
00061                      "      ...\n"
00062                      "    </my_controller>\n"
00063                      "    ...\n"
00064                      "  </controllers>\n\n"
00065                      "OPTIONAL XML CONFIGURATION\n\n"
00066                      "None for the time being.\n",
00067                      "Usable");
00068    
00069 }
```

---

Generated on 10 Jul 2018 for ARGoS by 
 1.6.1
